# Supplementary material for: Implication of Hepsin from Primary Tumor in the Prognosis of Colorectal Cancer Patients
Source: Cancers (Basel). 2022 Jun 24;14(13):3106. doi: 10.3390/cancers14133106 (PMC9264764; doi:10.3390/cancers14133106)
Supplement: Supplementary file 1 [file cancers-14-03106-s001.zip › Supplementary material 1.pdf]

Table S1. Percentage of patients in the overall, localized and metastatic cohort with different intensities of Hepsin staining in each of the discrete clinical and histopathological categories recorded. The *p*-values of Pearson's Chi-square correlations performed between hepsin and these variables are shown. HPN: Hepsin; N (%): Number and percentage of patients; ECOG: Eastern Cooperative Oncology Group; TNM II: T-stage 3/4, N-stage 0, M-stage 0; TNM III: T-stage 1/2/3/4, N-stage 1/2, M-stage 0; TNM IV: T-stage 1/2/3/4, N-stage 0/1/2, M-stage 1; T-stage: size and extension of the primary tumor; N-stage: tumor involvement of nearby lymph nodes; *: significant *p*-value.

**Overall cohort**	HPN staining intensity			p-value from Chi-square test
Clinical/histopathological variables correlated with HPN	Low	Medium	High	
Sex				0.819
Men: N (%)	39 (20.9)	66 (35.3)	82 (43.9)	
Women: N (%)	22 (22.0)	38 (38.0)	40 (40.0)	
ECOG at diagnosis				0.736
0: N (%)	23 (20.0)	48 (41.7)	44 (38.3)	
1: N (%)	27 (22.1)	40 (32.8)	55 (45.1)	
2: N (%)	5 (15.2)	11 (33.3)	17 (51.5)	
≥3: N (%)	1 (25.0)	1 (25.0)	2 (50.0)	
Primary tumor site				0.551
Ascending colon: N (%)	19 (22.9)	31 (37.3)	33 (39.8)	
Descending colon: N (%)	1 (7.1)	5 (35.7)	8 (57.1)	
Transverse colon: N (%)	3 (20.0)	8 (53.3)	4 (26.7)	
Sigmoid colon: N (%)	12 (18.5)	24 (36.9)	29 (44.6)	
Rectal: N (%)	26 (26.3)	30 (30.3)	43 (43.4)	
Number of chemotherapy lines				0.231
1: N (%)	11 (21.2)	20 (38.5)	21 (40.4)	
2: N (%)	11 (28.2)	10 (25.6)	18 (46.2)	
3: N (%)	5 (14.3)	17 (48.6)	13 (37.1)	
4: N (%)	3 (20.0)	4 (26.7)	8 (53.3)	
≥5: N (%)	0 (0.0)	9 (52.9)	8 (47.1)	
RAS mutations				0.574
Yes: N (%)	17 (17.3)	38 (38.8)	43 (43.9)	
No: N (%)	15 (23.1)	21 (32.3)	29 (44.6)	
BRAF mutations				0.579
Yes: N (%)	2 (33.3)	1 (16.7)	3 (50.0)	
No: N (%)	27 (20.5)	47 (35.6)	58 (43.9)	
Microsatellite instability				0.172
Yes: N (%)	2 (13.3)	3 (20.0)	10 (66.7)	
No: N (%)	26 (21.8)	44 (37.0)	49 (41.2)	
Histologic grade				<0.001*
Well differentiated: N (%)	22 (17.3)	55 (43.3)	50 (39.4)	
Moderately differentiated: N (%)	14 (16.5)	26 (30.6)	45 (52.9)	

Poorly differentiated: N (%)	13 (46.4)	4 (14.3)	11 (39.3)	
Lymphovascular invasion				0.656
No: N (%)	21 (21.9)	35 (36.5)	40 (41.7)	
Yes: N (%)	21 (17.1)	46 (37.4)	56 (45.5)	
Perineural invasion				0.633
No: N (%)	31 (19.4)	56 (35.0)	73 (45.6)	
Yes: N (%)	10 (17.5)	24 (42.1)	23 (40.4)	
Presence of tumor deposits distant to the primary tumor				0.197
No: N (%)	36 (20.6)	62 (35.4)	77 (44.0)	
Yes: N (%)	5 (12.5)	20 (50.0)	15 (37.5)	
Tumor stages: TNM II vs III vs IV				0.727
II: N (%)	12 (21.8)	19 (34.5)	24 (43.6)	
III: N (%)	14 (15.6)	36 (40.0)	40 (44.4)	
IV: N (%)	27 (22.9)	40 (33.9)	51 (43.2)	
Tumor stages: TNM II-III vs IV				0.750
II-III: N (%)	34 (20.1)	64 (37.9)	71 (42.0)	
IV: N (%)	27 (22.9)	40 (33.9)	51 (43.2)	
T-stage				0.110
T1: N (%)	2 (25.0)	2 (25.0)	4 (50.0)	
T2: N (%)	3 (17.6)	3 (17.6)	11 (64.7)	
T3: N (%)	23 (16.1)	53 (37.1)	67 (46.9)	
T4: N (%)	12 (25.0)	23 (47.9)	13 (27.1)	
N-stage				0.611
N0: N (%)	16 (21.3)	27 (36.0)	32 (42.7)	
N1: N (%)	16 (19.5)	33 (40.2)	33 (40.2)	
N2: N (%)	7 (12.3)	21 (36.8)	29 (50.9)	

**Localized cohort**	HPN staining intensity			p-value from Chi-square test
Clinical/histopathological variables correlated with HPN	Low	Medium	High	
Sex				0.902
Men: N (%)	21 (19.3)	41 (37.6)	47 (43.1)	
Women: N (%)	13 (21.7)	23 (38.3)	24 (40.0)	
ECOG at diagnosis				0.334
0: N (%)	15 (17.6)	38 (44.7)	32 (37.6)	
1: N (%)	15 (22.4)	21 (31.3)	31 (46.3)	
2: N (%)	2 (18.2)	2 (18.2)	7 (63.6)	
≥3: N (%)	0 (0.0)	1 (100.0)	0 (0.0)	
Primary tumor site				0.513
Ascending colon: N (%)	12 (25.0)	16 (33.3)	20 (41.7)	
Descending colon: N (%)	1 (11.1)	3 (33.3)	5 (55.6)	
Transverse colon: N (%)	1 (10.0)	6 (60.0)	3 (30.0)	
Sigmoid colon: N (%)	4 (11.4)	16 (45.7)	15 (42.9)	
Rectal: N (%)	16 (25.0)	21 (32.8)	27 (42.2)	
Number of chemotherapy lines				0.275
1: N (%)	5 (22.7)	6 (27.3)	11 (50.0)	
2: N (%)	0 (0.0)	5 (50.0)	5 (50.0)	
3: N (%)	0 (0.0)	6 (50.0)	6 (50.0)	
4: N (%)	0 (0.0)	1 (33.3)	2 (66.6)	
≥5: N (%)	1 (25.0)	3 (75.0)	0 (0.0)	

RAS mutations				0.381
Yes: N (%)	2 (5.7)	16 (45.7)	17 (48.6)	
No: N (%)	4 (16.7)	9 (37.5)	11 (45.8)	
BRAF mutations				0.168
Yes: N (%)	1 (50.0)	0 (0.0)	1 (50.0)	
No: N (%)	5 (9.8)	21 (41.2)	25 (49.0)	
Microsatellite instability				0.218
Yes: N (%)	2 (15.4)	2 (15.4)	9 (69.2)	
No: N (%)	13 (17.1)	29 (38.2)	34 (44.7)	
Histologic grade				0.057
Well differentiated: N (%)	17 (18.9)	33 (36.7)	40 (44.4)	
Moderately differentiated: N (%)	10 (18.9)	23 (43.4)	20 (37.7)	
Poorly differentiated: N (%)	5 (50.0)	0 (0.0)	5 (50.0)	
Lymphovascular invasion				0.626
No: N (%)	16 (21.3)	28 (37.3)	31 (41.3)	
Yes: N (%)	12 (15.8)	28 (36.8)	36 (47.4)	
Perineural invasion				0.653
No: N (%)	23 (19.7)	44 (37.6)	50 (42.7)	
Yes: N (%)	5 (15.2)	11 (33.3)	17 (51.5)	
Presence of tumor deposits distant to the primary tumor				0.156
No: N (%)	26 (20.5)	46 (36.2)	55 (43.3)	
Yes: N (%)	2 (10.0)	9 (45.0)	9 (45.0)	
Tumor stages: TNM II vs III				0.601
II: N (%)	12 (21.8)	19 (34.5)	24 (43.6)	
III: N (%)	14 (15.6)	36 (40.0)	40 (44.4)	
T-stage				0.256
T1: N (%)	2 (40.0)	1 (20.0)	2 (40.0)	
T2: N (%)	3 (20.0)	3 (20.0)	9 (60.0)	
T3: N (%)	17 (16.0)	40 (37.7)	49 (46.2)	
T4: N (%)	5 (23.8)	11 (52.4)	5 (23.8)	
N-stage				0.357
N0: N (%)	12 (21.8)	19 (34.5)	24 (43.6)	
N1: N (%)	12 (20.3)	24 (40.7)	23 (39.0)	
N2: N (%)	2 (6.5)	12 (38.7)	17 (54.8)	
**Metastatic cohort**	HPN staining intensity			p-value from Chi-square test
Clinical/histopathological variables correlated with HPN	Low	Medium	High	
Sex				0.827
Men: N (%)	18 (23.1)	25 (32.1)	35 (44.9)	
Women: N (%)	9 (22.5)	15 (37.5)	16 (40.0)	
ECOG at diagnosis				0.814
0: N (%)	8 (26.7)	10 (33.3)	12 (40.0)	
1: N (%)	12 (21.8)	19 (34.5)	24 (43.6)	
2: N (%)	3 (13.6)	9 (40.9)	10 (45.5)	
≥3: N (%)	1 (33.3)	0 (0.0)	2 (66.7)	
Primary tumor site				0.645
Ascending colon: N (%)	7 (20.0)	15 (42.9)	13 (37.1)	
Descending colon: N (%)	0 (0.0)	2 (40.0)	3 (60.0)	
Transverse colon: N (%)	2 (40.0)	2 (40.0)	1 (20.0)	

Sigmoid colon: N (%)	8 (26.7)	8 (26.7)	14 (46.7)	
Rectal: N (%)	10 (28.6)	9 (25.7)	16 (45.7)	
Liver metastasis at diagnosis				0.810
Yes: N (%)	20 (23.8)	27 (32.1)	37 (44.0)	
No: N (%)	7 (20.6)	13 (38.2)	14 (41.2)	
Peritoneal metastasis at diagnosis				0.110
Yes: N (%)	11 (34.4)	7 (21.9)	14 (43.8)	
No: N (%)	16 (18.6)	33 (38.4)	37 (43.0)	
Lung metastasis at diagnosis				0.245
Yes: N (%)	10 (32.3)	11 (35.5)	10 (32.3)	
No: N (%)	17 (19.5)	29 (33.3)	41 (47.1)	
>1 metastasis site at diagnosis				0.023*
Yes: N (%)	16 (34.8)	16 (34.8)	14 (30.4)	
No: N (%)	11 (15.3)	24 (33.3)	37 (51.4)	
Number of chemotherapy lines				0.106
1: N (%)	6 (20.0)	14 (46.7)	10 (33.3)	
2: N (%)	11 (37.9)	5 (17.2)	13 (44.8)	
3: N (%)	5 (21.7)	11 (47.8)	7 (30.4)	
4: N (%)	3 (25.0)	3 (25.0)	6 (50.0)	
≥5: N (%)	0 (0.0)	6 (46.2)	7 (53.8)	
RAS mutations				0.829
Yes: N (%)	15 (23.8)	22 (34.9)	26 (41.3)	
No: N (%)	11 (26.8)	12 (29.3)	18 (43.9)	
BRAF mutations				0.929
Yes: N (%)	1 (25.0)	1 (25.0)	2 (50.0)	
No: N (%)	22 (27.2)	26 (32.1)	33 (40.7)	
Microsatellite instability				0.654
Yes: N (%)	0 (0.0)	1 (50.0)	1 (50.0)	
No: N (%)	13 (30.2)	15 (34.9)	15 (34.9)	
Histologic grade				<0.001*
Well differentiated: N (%)	5 (13.5)	22 (59.5)	10 (27.0)	
Moderately differentiated: N (%)	4 (12.5)	3 (9.4)	25 (78.1)	
Poorly differentiated: N (%)	8 (44.4)	4 (22.2)	6 (33.3)	
Lymphovascular invasion				0.626
No: N (%)	16 (21.3)	28 (37.3)	31 (41.3)	
Yes: N (%)	12 (15.8)	28 (36.8)	36 (47.4)	
Perineural invasion				0.653
No: N (%)	23 (19.7)	44 (37.6)	50 (42.7)	
Yes: N (%)	5 (15.2)	11 (33.3)	17 (51.5)	
Presence of tumor deposits distant to the primary tumor				0.156
No: N (%)	26 (20.5)	46 (36.2)	55 (43.3)	
Yes: N (%)	2 (10.0)	9 (45.0)	9 (45.0)	
T-stage				0.399
T1: N (%)	0 (0.0)	1 (33.3)	2 (66.6)	
T2: N (%)	0 (0.0)	0 (0.0)	2 (100.0)	
T3: N (%)	6 (16.2)	13 (35.1)	18 (48.6)	
T4: N (%)	7 (25.9)	12 (44.4)	8 (29.6)	
N-stage				0.993
N0: N (%)	4 (20.0)	8 (40.0)	8 (40.0)	
N1: N (%)	4 (17.4)	9 (39.1)	10 (43.5)	

N2: N (%)	5 (19.2)	9 (34.6)	12 (46.2)	
