# Supplementary material for: Implication of Hepsin from Primary Tumor in the Prognosis of Colorectal Cancer Patients
Source: Cancers (Basel). 2022 Jun 24;14(13):3106. doi: 10.3390/cancers14133106 (PMC9264764; doi:10.3390/cancers14133106)
Supplement: Supplementary file 1 [file cancers-14-03106-s001.zip › Supplementary material 2.pdf]

Table S2. Correlations between Hepsin staining intensity and time-to-event variables. The results of these correlations are shown for the overall cohort and localized and metastatic patients separately. HPN: Hepsin; N: number of patients for each hepsin staining intensity; CI: confidence interval; LL: lower limit; UL: upper limit; tft: Log-rank test for trends; Stat: statistical value; df: degrees of freedom; *: significant *p*-value. † Follow-up limit reached.

**Overall cohort**

| HPN staining intensity | N | Death events | 5-year overall survival (%) | Standard error (%) | LL CI 95% (%) | UL CI 95% (%) | p-value tft |
|---|---|---|---|---|---|---|---|
| High | 121 | 60 | 41 | 6 | 31 | 55 | 0.40 |
| Medium | 104 | 52 | 59 | 6 | 49 | 71 | |
| Low | 61 | 29 | 55 | 7 | 43 | 71 | |

| HPN staining intensity | N | Thrombosis events | 5-year cumulative incidence of thrombosis (%) | Standard error (%) | LL CI 95% (%) | UL CI 95% (%) | p-value tft | Gray´s test | | |
|---|---|---|---|---|---|---|---|---|---|---|
| | | | | | | | | Stat | df | p-value |
| High | 121 | 31 | 30 | 5 | 20 | 38 | 0.61 | 2.60 | 2 | 0.27 |
| Medium | 104 | 24 | 26 | 5 | 15 | 35 | | | | |
| Low | 61 | 10 | 15 | 5 | 5 | 24 | | | | |

**Localized cohort**

| HPN staining intensity | N | Death events | 5-year overall survival (%) | Standard error (%) | LL CI 95% (%) | UL CI 95% (%) | p-value tft |
|---|---|---|---|---|---|---|---|
| High | 71 | 16 | 63 | 9 | 48 | 83 | 0.67 |
| Medium | 64 | 22 | 70 | 7 | 58 | 86 | |
| Low | 34 | 8 | 81 | 8 | 67 | 99 | |

| HPN staining intensity | N | Metastatic relapse events | 5-year disease-free survival (%) | Standard error (%) | LL CI 95% (%) | UL CI 95% (%) | p-value tft |
|---|---|---|---|---|---|---|---|
| High | 71 | 26 | 51 | 8 | 37 | 71 | 0.16 |
| Medium | 64 | 22 | 59 | 7 | 46 | 75 | |
| Low | 34 | 6 | 73 | 10 | 56 | 96 | |

| HPN staining intensity | N | Thrombosis events | 5-year cumulative incidence of thrombosis (%) | Standard error (%) | LL CI 95% (%) | UL CI 95% (%) | p-value tft | Gray´s test | | |
|---|---|---|---|---|---|---|---|---|---|---|
| | | | | | | | | Stat | df | p-value |
| High | 71 | 16 | 23 | 5 | 12 | 33 | 0.038* | 19.38 | 2 | 0.009* |
| Medium | 64 | 13 | 22 | 6 | 9 | 33 | | | | |
| Low | 34 | 1 | 0 | 0 | 0 | 0 | | | | |

**Metastatic cohort**

| HPN staining intensity | N | Death events | 5-year overall survival (%) | Standard error (%) | LL CI 95% (%) | UL CI 95% (%) | p-value tft |
|---|---|---|---|---|---|---|---|
| High | 50 | 44 | 18 | 6 | 9 | 34 | 0.21 |
| Medium | 40 | 30 | 41 | 8 | 28 | 61 | |
| Low | 27 | 21 | 24 | 8 | 12 | 48 | |

| HPN staining intensity | N | Progression events | 4-year[†] progression-free survival (%) | Standard error (%) | LL CI 95% (%) | UL CI 95% (%) | p-value tft |
|---|---|---|---|---|---|---|---|
| High | 44 | 43 | 5 | 3 | 1 | 18 | 0.20 |
| Medium | 38 | 35 | 11 | 5 | 4 | 27 | |
| Low | 25 | 24 | 4 | 4 | 1 | 27 | |

| HPN staining intensity | N | Thrombosis events | 5-year cumulative incidence of thrombosis (%) | Standard error (%) | LL CI 95% (%) | UL CI 95% (%) | p-value tft | Gray´s test | | |
|---|---|---|---|---|---|---|---|---|---|---|
| | | | | | | | | Stat | df | p-value |
| High | 50 | 15 | 43 | 9 | 22 | 58 | 0.23 | 0.83 | 2 | 0.66 |
| Medium | 40 | 11 | 31 | 9 | 11 | 46 | | | | |
| Low | 27 | 9 | 35 | 11 | 13 | 52 | | | | |
