# Supplementary material for: Implication of Hepsin from Primary Tumor in the Prognosis of Colorectal Cancer Patients
Source: Cancers (Basel). 2022 Jun 24;14(13):3106. doi: 10.3390/cancers14133106 (PMC9264764; doi:10.3390/cancers14133106)
Supplement: Supplementary file 1 [file cancers-14-03106-s001.zip › Supplementary material 3.pdf]

Table S3. Correlations between Hepsin staining intensity and continuous clinical-histopathological variables. The results of these correlations are shown for the overall cohort and localized and metastatic patients separately. HPN: Hepsin; CEA: carcinoembryonic antigen; LDH: lactate dehydrogenase.

**Overall cohort**	HPN staining intensity			p-value from Kruskal-Wallis test
Clinical/histopathological variables correlated with HPN	Low	Medium	High	
Age at diagnosis (years): Mean; standard error of mean	62.5; 1.8	62.9; 1.2	66.1; 0.9	0.176
CEA (ng/ml): Mean; standard error of mean	49.1; 20.4	30.7; 8.9	22.1; 4.7	0.334
Leukocytes (x10^3/µl): Mean; standard error of mean	8.0; 0.3	8.6; 0.4	8.3; 0.3	0.573
Platelets (x10^3/µl): Mean; standard error of mean	307.0; 17.7	281.0; 12.7	278.0; 10.9	0.368
LDH (U/l): Mean; standard error of mean	420.0; 76.3	497.0; 63.1	578.0; 107.0	0.828
Number of tumor deposits distant to the primary tumor: Mean; standard error of mean	1.0; 0.7	0.8; 0.2	0.4; 0.1	0.508
**Localized cohort**	HPN staining intensity			p-value from Kruskal-Wallis test
Clinical/histopathological variables correlated with HPN	Low	Medium	High	
Age at diagnosis (years): Mean; standard error of mean	60.9; 2.3	62.3; 1.4	65.4; 1.1	0.223
CEA (ng/ml): Mean; standard error of mean	5.8; 2.7	6.9; 1.9	6.6; 1.7	0.315
Leukocytes (x10^3/µl): Mean; standard error of mean	7.5; 0.4	7.8; 0.4	7.7; 0.4	0.964
Platelets (x10^3/µl): Mean; standard error of mean	304.0; 27.8	279.0; 14.7	271.0; 14.6	0.684
LDH (U/l): Mean; standard error of mean	310.0; 35.1	338.0; 18.1	330.0; 17.4	0.809
Number of tumor deposits distant to the primary tumor: Mean; standard error of mean	1.1; 1.1	0.5; 0.2	0.3; 0.1	0.664
**Metastatic cohort**	HPN staining intensity			p-value from Kruskal-Wallis test
Clinical/histopathological variables correlated with HPN	Low	Medium	High	
Age at diagnosis (years): Mean; standard error of mean	64.5; 2.7	63.9; 2.2	66.9; 1.5	0.727
CEA (ng/ml): Mean; standard error of mean	106.0; 44.4	72.5; 22.5	48.3; 11.0	0.614
Leukocytes (x10^3/µl): Mean; standard error of mean	8.7; 0.5	9.9; 0.6	9.1; 0.6	0.481

Platelets (x10^3/µl): Mean; standard error of mean	311.0; 19.2	284.0; 23.2	289.0; 16.2	0.527
LDH (U/l): Mean; standard error of mean	496.0; 124.0	685.0; 122.0	939.0; 231.0	0.532
Number of tumor deposits distant to the primary tumor: Mean; standard error of mean	0.8; 0.4	1.4; 0.5	0.5; 0.2	0.417
