# Supplementary material for: Implication of Hepsin from Primary Tumor in the Prognosis of Colorectal Cancer Patients
Source: Cancers (Basel). 2022 Jun 24;14(13):3106. doi: 10.3390/cancers14133106 (PMC9264764; doi:10.3390/cancers14133106)
Supplement: Supplementary file 1 [file cancers-14-03106-s001.zip › Supplementary material 4.pdf]

Table S4. Clinical scenario of localized patients who suffered from thrombosis during follow-up. For each patient, a series of characteristics potentially underlying the origin of the thrombotic event are shown. ID: identifier; PE: pulmonary embolism; DVT: deep vein thrombosis; NOS: not otherwise specified; NA: not applicable; CAPOX: Capecitabine + Oxaliplatin; FUOX: Fluorouracil + Oxaliplatin; 5FU: 5-Fluorouracil; FOLFIRI: Folinic acid + Fluorouracil + Irinotecan; SNG + NTP: Nasogastric tube+parenteral nutrition.

Patient ID	Location of thrombosis	Months until thrombosis since tumor diagnosis	Patient suffers metastasis relapse before thrombosis?	Months between relapse and thrombosis	Hemostasis-related background before tumor diagnosis	Chemotherapy treatment before thrombosis	Months between chemotherapy and thrombosis	Particular incidents of each patient
1	Lower extremity, NOS	11.27	No	NA	Arterial hypertension/ dyslipidemia/ supraventricular tachycardia	Adjuvant CAPOX	8.83	At diagnosis of the colorectal tumor, the patient also had a spiculated nodule in the left upper lobe (it could be a parenchymal scar vs a primary neoplasm) that persisted in August 2018, four months before thrombosis
2	DVT, NOS	20.03	No	NA	NA	NA	NA	NA
3	Head & neck	26.40	No	NA	NA	NA	NA	Patient was admitted to the hospital for

								ileostomy closure with transit reconstruction when he had the thrombosis
4	Lower extremity, NOS	6.47	No	NA	NA	Adjuvant Capecitabine	3.47	NA
5	Splanchnic	4.90	No	NA	Dyslipidemia	Adjuvant Capecitabine	3.87	NA
6	Femoral + PE	2.03	No	NA	NA	Neoadjuvant radiotherapy + Capecitabine	1.20	At the start of neoadjuvant treatment, patient suffered polymicrobial right sternoclavicular septic arthritis, with previous trauma in the affected area
7	PE, NOS	0.47	No	NA	NA	NA	NA	NA
8	Splanchnic	8.77	No	NA	NA	Adjuvant CAPOX	2.67	NA
9	Femoral	6.87	No	NA	Arterial hypertension	Adjuvant CAPOX	3.97	NA
10	PE, NOS	2.10	No	NA	Arterial hypertension/ overweight	Adjuvant CAPOX	0.90	Two months before thrombosis, a right hemicolectomy was performed, and after the operation, there was a prolonged ileus that was resolved with

								SNG+NTP treatment
11	PE, NOS	5.17	No	NA	NA	Adjuvant CAPOX	3.13	NA
12	Calf vein	2.00	No	NA	Arterial hypertension	Adjuvant CAPOX	1.00	NA
13	Femoral	4.03	No	NA	NA	Adjuvant CAPOX	3.03	NA
14	PE, NOS	9.10	No	NA	Dyslipidemia	Adjuvant CAPOX+FUOX	6.50	NA
15	Lower extremity, NOS	2.13	No	NA	NA	Adjuvant Capecitabine	0.47	NA
16	Catheter-related + PE	0.53	No	NA	NA	NA	NA	Patient had thrombosis the same month he had rectal surgery
17	PE, NOS	4.07	Yes	2.30	Arterial hypertension/ non-insulinized type-II diabetes mellitus	Oxaliplatin+Xeloda	2.13	One month before thrombosis, it was detected a doubtful uterine myoma respecting its status as a primary or metastatic tumor
18	Femoral	33.43	Yes	3.03	Arterial hypertension/ dyslipidemia/ type-II diabetes mellitus/ dyspnea on	Capecitabine+Avastin	2.47	NA

					exertion			
19	Femoral	47.20	Yes	24.30	NA	5FU+Avastin	5.57	NA
20	PE, NOS	35.10	Yes	9.23	Dyslipidemia/ ischemic heart disease	Oxaliplatin+5FU+Bevacizumab	11.40	NA
21	PE, NOS	12.37	Yes	0.10	Dyslipidemia/ chronic arthritis in lower extremities	NA	NA	Patient was admitted to the hospital when he suffered thrombosis
22	Lower extremity, NOS	22.33	Yes	3.00	NA	Irinotecan+Cetuximab	3.07	NA
23	Splanchnic	9.77	Yes	1.70	Patient walks with help but with difficulty due to shortening of the right leg	NA	NA	NA
24	Head & neck	114.90	Yes	58.40	Arterial hypertension/ generalized osteoarthritis	NA	NA	The day when thrombosis was detected, patient had previously fallen twice at home
25	PE, NOS	18.67	Yes	8.50	NA	FOLFIRI+Avastin/Capecitabine+Avastin	8.53	NA
26	Splanchnic	62.17	Yes	14.47	Arterial hypertension/ dyslipidemia/	FOLFIRI	8.43	NA

					embolic cardiovascular accident			
27	Lower extremity, NOS	28.03	Yes	11.80	NA	FOLFIRI+Aflibercept	6.73	It was a patient treated with low molecular weight heparin who stopped taking it due to episodes of peristomal bleeding prior to thrombosis
28	Splanchnic	3.60	Yes	0.27	Thoracic aortic aneurysm/ knee osteoarthritis	NA	NA	NA
29	PE, NOS	23.37	Yes	8.10	Arterial hypertension/ polycythemia vera	NA	NA	NA
30	Splanchnic	63.77	Yes	49.60	Arterial hypertension/ dyslipidemia	Oxaliplatin+5FU+Bevacizumab	36.97	Postoperative thrombosis
