# Supplementary material for: Implication of Hepsin from Primary Tumor in the Prognosis of Colorectal Cancer Patients
Source: Cancers (Basel). 2022 Jun 24;14(13):3106. doi: 10.3390/cancers14133106 (PMC9264764; doi:10.3390/cancers14133106)
Supplement: Supplementary file 1 [file cancers-14-03106-s001.zip › Supplementary material 5.pdf]

Table S5. Multivariable models for overall survival, progression-free survival and cumulative incidence of thrombosis in metastatic patients at diagnosis: The risk of death is calculated according to Hepsin staining intensity, adjusting this calculation by adding different covariates to the model. The risk of progression is calculated according to Hepsin staining intensity, adjusting this calculation by adding different covariates to the model. The risk of thrombosis is calculated according to Hepsin staining intensity, adjusting this calculation by adding different covariates to the model. OS: overall survival; Coef: Cox regression coefficient; exp (coef): hazard ratio; CI: confidence interval; LL: lower limit; UL: upper limit; se (coef): standard error of Cox regression coefficient; *p*: *p*-value; HPN: Hepsin; ECOG: Eastern Cooperative Oncology Group; PFS: progression-free survival after first-line chemotherapy; *: significant *p*-value.

**Multivariate Cox regression for OS**						
**Regressions**	**Coef**	**exp(coef)**	**LL CI 95% of exp(coef)**	**UL CI 95% of exp(coef)**	**se(coef)**	**p**
HPN Medium	0.11	1.11	0.64	1.95	0.29	0.71
HPN High	-0.05	0.95	0.50	1.80	0.33	0.87
HPN Low (reference)	-	-	-	-	-	-
Moderately differentiated histological grade	0.27	1.31	0.76	2.26	0.28	0.34
Poorly differentiated histological grade	1.28	3.59	1.88	6.83	0.33	0.0001*
Well differentiated histological grade (reference)	-	-	-	-	-	-
ECOG 1	0.33	1.39	0.81	2.38	0.27	0.23
ECOG 2	0.84	2.31	1.22	4.38	0.33	0.01*
ECOG ≥3	2.50	12.23	3.07	48.70	0.70	0.0004*
ECOG 0 (reference)	-	-	-	-	-	-

>1 metastasis site	-0.28	0.76	0.40	1.42	0.32	0.39
1 metastasis site (reference)	-	-	-	-	-	-
Peritoneal involvement	0.13	1.14	0.57	2.25	0.35	0.71
No peritoneal involvement (reference)	-	-	-	-	-	-
Lung involvement	0.21	1.23	0.65	2.31	0.32	0.52
No lung involvement (reference)	-	-	-	-	-	-
Liver involvement	0.16	1.17	0.61	2.25	0.34	0.64
No liver involvement (reference)	-	-	-	-	-	-

**Multivariate Cox regression for PFS**						
**Regressions**	**Coef**	**exp(coef)**	**LL CI 95% of exp(coef)**	**UL CI 95% of exp(coef)**	**se(coef)**	**p**
HPN Medium	0.14	1.15	0.64	2.04	0.29	0.64
HPN High	0.05	1.05	0.59	1.89	0.30	0.86
HPN Low (reference)	-	-	-	-	-	-
Moderately differentiated histological grade	0.47	1.60	0.94	2.70	0.27	0.08
Poorly differentiated histological grade	1.07	2.91	1.62	5.25	0.30	0.0004*
Well differentiated histological grade (reference)	-	-	-	-	-	-
ECOG 1	-0.21	0.81	0.50	1.32	0.25	0.39
ECOG 2	0.34	1.41	0.75	2.66	0.32	0.29
ECOG ≥3	0.94	2.56	0.65	10.12	0.70	0.18

ECOG 0 (reference)	-	-	-	-	-	-
>1 metastasis site	-0.16	0.85	0.45	1.64	0.33	0.63
1 metastasis site (reference)	-	-	-	-	-	-
Peritoneal involvement	0.31	1.36	0.62	3.00	0.40	0.45
No peritoneal involvement (reference)	-	-	-	-	-	-
Lung involvement	0.20	1.22	0.60	2.50	0.36	0.58
No lung involvement (reference)	-	-	-	-	-	-
Liver involvement	0.07	1.07	0.50	2.28	0.39	0.86
No liver involvement (reference)	-	-	-	-	-	-

**Multivariate Fine&Gray regression for cumulative incidence of thrombosis**						
**Regressions**	**Coef**	**exp(coef)**	**LL CI 95% of exp(coef)**	**UL CI 95% of exp(coef)**	**se(coef)**	**p**
HPN Medium	-0.15	0.86	0.30	2.47	0.54	0.78
HPN High	-0.08	0.92	0.37	2.33	0.47	0.87
HPN Low (reference)	-	-	-	-	-	-
Moderately differentiated histological grade	-0.18	0.84	0.34	2.05	0.46	0.69
Poorly differentiated histological grade	1.05	2.85	1.11	7.33	0.48	0.029*
Well differentiated histological grade (reference)	-	-	-	-	-	-
Lymphovascular invasion	0.04	1.04	0.47	2.30	0.41	0.93
Absent lymphovascular invasion (reference)	-	-	-	-	-	-
>1 metastasis site	-0.56	0.57	0.20	1.67	0.55	0.31
1 metastasis site (reference)	-	-	-	-	-	-

Peritoneal involvement	-0.32	0.73	0.25	2.12	0.55	0.56
No peritoneal involvement (reference)	-	-	-	-	-	-
Lung involvement	0.74	2.09	0.63	6.92	0.61	0.23
No lung involvement (reference)	-	-	-	-	-	-
Liver involvement	0.24	1.27	0.44	3.68	0.54	0.66
No liver involvement (reference)	-	-	-	-	-	-
