# Supplementary material for: Implication of Hepsin from Primary Tumor in the Prognosis of Colorectal Cancer Patients
Source: Cancers (Basel). 2022 Jun 24;14(13):3106. doi: 10.3390/cancers14133106 (PMC9264764; doi:10.3390/cancers14133106)
Supplement: Supplementary file 1 [file cancers-14-03106-s001.zip › Supplementary material 6.pdf]

Table S6. Correlations between Hepsin staining intensity and time-to-event variables in metastatic patients with mutated RAS. Upper table shows correlations based on the Kaplan–Meier estimator and the Log-rank for trends and Gray's tests. Lower tables show Cox and Fine and Gray multivariate models. HPN: Hepsin; N: number of patients for each hepsin level; CI: confidence interval; LL: lower limit; UL: upper limit; tft: Log-rank test for trends; Stat: statistical value; df: degrees of freedom; OS: overall survival; Coef: Cox regression coefficient; exp (coef): hazard ratio; se (coef): standard error of Cox regression coefficient; p: p-value; ECOG: Eastern Cooperative Oncology Group; PFS: progression-free survival after first-line chemotherapy; *: significant *p*-value. † Follow-up limit reached.

**HPN staining intensity**	**N**	**Death events**	**5-year overall survival (%)**	**Standard error (%)**	**LL CI 95% (%)**	**UL CI 95% (%)**	**p-value tft**
High	26	25	5	5	1	31	0.09
Medium	22	16	39	11	23	67	
Low	15	11	23	11	9	61	

**HPN staining intensity**	**N**	**Progression events**	**4-year† progression-free survival (%)**	**Standard error (%)**	**LL CI 95% (%)**	**UL CI 95% (%)**	**p-value tft**
High	23	22	9	6	2	33	0.42
Medium	22	20	9	6	2	34	
Low	15	14	7	6	1	44	

**HPN staining intensity**	**N**	**Thrombosis events**	**4-year† cumulative incidence of thrombosis (%)**	**Standard error (%)**	**LL CI 95% (%)**	**UL CI 95% (%)**	**p-value tft**	**Gray´s test**		
								**Stat**	**df**	**p-value**
High	26	10	52	12	21	71	0.48	0.69	2	0.71
Medium	22	8	30	10	6	47				
Low	15	6	43	14	9	64				

Multivariate Cox regression for PFS						
**Regressions**	**Coef**	**exp(coef)**	**LL CI 95% of exp(coef)**	**UL CI 95% of exp(coef)**	**se(coef)**	**p**
HPN Medium	-0.05	0.95	0.44	2.05	0.39	0.90
HPN High	0.09	1.10	0.45	2.68	0.46	0.84
HPN Low (reference)	-	-	-	-	-	-
Moderately differentiated histological grade	0.48	1.61	0.67	3.84	0.44	0.28
Poorly differentiated histological grade	1.24	3.47	1.48	8.16	0.44	0.004*
Well differentiated histological grade (reference)	-	-	-	-	-	-
ECOG 1	-0.13	0.88	0.44	1.76	0.36	0.71
ECOG 2	0.25	1.28	0.53	3.07	0.45	0.58
ECOG 0 (reference)	-	-	-	-	-	-
>1 metastasis site	1.32	3.73	1.41	9.84	0.49	0.008*
1 metastasis site (reference)	-	-	-	-	-	-
Peritoneal involvement	-0.66	0.52	0.17	1.52	0.55	0.23
No peritoneal involvement (reference)	-	-	-	-	-	-
Lung involvement	-1.49	0.22	0.08	0.60	0.50	0.003*
No lung involvement (reference)	-	-	-	-	-	-
Liver involvement	-1.47	0.23	0.08	0.68	0.55	0.008*
No liver involvement (reference)	-	-	-	-	-	-

Multivariate Cox regression for OS						
**Regressions**	**Coef**	**exp(coef)**	**LL CI 95% of exp(coef)**	**UL CI 95% of exp(coef)**	**se(coef)**	**p**
HPN Medium	-0.21	0.81	0.31	2.13	0.49	0.67
HPN High	0.42	1.51	0.63	3.65	0.45	0.35
HPN Low (reference)	-	-	-	-	-	-
Moderately differentiated histological grade	0.72	2.05	0.92	4.56	0.41	0.08
Poorly differentiated histological grade	1.19	3.29	1.38	7.86	0.44	0.007*
Well differentiated histological grade (reference)	-	-	-	-	-	-
ECOG 1	-0.28	0.76	0.36	1.60	0.38	0.47
ECOG 2	1.02	2.76	1.07	7.17	0.49	0.037*
ECOG 0 (reference)	-	-	-	-	-	-
>1 metastasis site	0.89	2.44	0.94	6.38	0.49	0.07
1 metastasis site (reference)	-	-	-	-	-	-
Peritoneal involvement	-0.21	0.81	0.29	2.24	0.52	0.69
No peritoneal involvement (reference)	-	-	-	-	-	-
Lung involvement	-1.31	0.27	0.10	0.73	0.51	0.01*
No lung involvement (reference)	-	-	-	-	-	-
Liver involvement	-0.66	0.52	0.20	1.33	0.48	0.17
No liver involvement (reference)	-	-	-	-	-	-

Multivariate Fine&Gray regression for cumulative incidence of thrombosis						
**Regressions**	**Coef**	**exp(coef)**	**LL CI 95% of exp(coef)**	**UL CI 95% of exp(coef)**	**se(coef)**	**p**
HPN Medium	-0.58	0.56	0.18	1.78	0.59	0.33
HPN High	-0.65	0.52	0.16	1.71	0.61	0.28
HPN Low (reference)	-	-	-	-	-	-
Moderately differentiated histological grade	-0.53	0.59	0.16	2.21	0.68	0.43
Poorly differentiated histological grade	1.59	4.91	1.19	20.32	0.73	0.028*
Well differentiated histological grade (reference)	-	-	-	-	-	-
Lymphovascular invasion	-0.37	0.69	0.27	1.72	0.47	0.43
Absent lymphovascular invasion (reference)	-	-	-	-	-	-
>1 metastasis site	-1.84	0.16	0.01	1.88	1.26	0.14
1 metastasis site (reference)	-	-	-	-	-	-
Peritoneal involvement	0.78	2.19	0.22	21.31	1.16	0.50
No peritoneal involvement (reference)	-	-	-	-	-	-
Lung involvement	2.87	17.62	0.89	348.05	1.52	0.06
No lung involvement (reference)	-	-	-	-	-	-
Liver involvement	1.89	6.58	0.39	110.42	1.44	0.19
No liver involvement (reference)	-	-	-	-	-	-
